# Long‐term monitoring of dynamic changes in plasma EBV DNA for improved prognosis prediction of nasopharyngeal carcinoma

**DOI:** 10.1002/cam4.3669

**Published:** 2020-12-30

**Authors:** Wanxia Li, Jing Chen, Bijun Liang, Zonghua Li, Junzheng Li, Xiaofei Yuan, Shuting Wu, Fangfang Zeng, Xinyu Peng, Yanfei Li, Juan Lu, Feipeng Zhao, Xiong Liu

**Affiliations:** ^1^ Department of Otolaryngology‐Head and Neck Surgery Nanfang Hospital Southern Medical University Guangzhou Guangdong China; ^2^ School of Traditional Chinese Medicine Southern Medical University Guangzhou Guangdong China; ^3^ Department of Otolaryngology‐Head and Neck Surgery The Affiliated Hospital of Southwest Medical University Southwest Medical University Luzhou Sichuan China; ^4^ Department of Otolaryngology 942 Hospital of the Chinese People's Liberation Army Yinchuan Ningxia China; ^5^ Department of Otolaryngology‐Head and Neck Surgery Guangzhou Red Cross Hospital Jinan University Guangzhou Guangdong China

**Keywords:** dynamic change, EBV DNA, nasopharyngeal carcinoma, plasma, prognosis

## Abstract

**Background:**

This study was performed to investigate whether long‐term monitoring of dynamic changes in plasma Epstein‐Barr virus (EBV) DNA could improve prognosis prediction of nasopharyngeal carcinoma (NPC).

**Materials and methods:**

About 1077 nonmetastatic NPC patients were recruited to retrospectively analyze the prognostic value of plasma EBV DNA load pretreatment and 3, 12, 24, and 36 months posttreatment. We also examined the prognostic value of dynamic changes in plasma EBV DNA at various time points.

**Results:**

Patients with plasma EBV DNA load above optimal pre‐ and posttreatment cut‐offs had significantly worse five‐year progression‐free survival, distant metastasis‐free survival, locoregional relapse‐free survival, and overall survival (OS) at all‐time points, excluding only OS at 36 months posttreatment due to limited mortalities. Patients with persistently undetectable plasma EBV DNA at the first four time points had the best prognosis, followed by those with positive detection pretreatment and consistently negative detection posttreatment, those with negative detection pretreatment and positive detection at one time point posttreatment, and those with positive detection pretreatment and at one time point posttreatment, whereas patients with positive detection at ≥2 time points posttreatment had the worst prognosis. Cox proportional hazard models identified the dynamic change pattern as an independent prognostic factor, and receiver operating characteristic curve analysis demonstrated that the dynamic change at four time point was more valuable than any single time point for predicting disease progression, distant metastasis, locoregional relapse, and mortality.

**Conclusions:**

Dynamic changes in plasma EBV DNA pre‐ and posttreatment could predict the long‐term survival outcome and provide accurate risk stratification in NPC.

## Graphical abstract

As previous studies only measured plasma Epstein‐Barr virus (EBV) DNA at limited time points, and data investigating the prognostic value of dynamic changes in plasma EBV DNA level for NPC are limited. In our study, we found that plasma EBV DNA load at multiple time points pre‐ and post‐ treatment was a significant predictor for NPC prognosis, and the dynamic change pattern in plasma EBV DNA at these time points could provide much accurate predictive value for NPC prognosis and risk stratification.

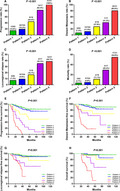

## 1 INTRODUCTION

Nasopharyngeal carcinoma (NPC) is a malignant head and neck tumor originating from the nasopharyngeal epithelia that is highly prevalent in Southeast Asia, especially in southern China.^1^ With the development of radiotherapy and chemotherapy regimens,^2^ the survival outcomes of NPC patients have greatly improved. However, about 5%–15% of NPC patients develop nasopharynx or regional lymph node recurrence after treatment, and up to 10%–30% patients develop distant metastasis.^3^ Therefore, identification of biomarkers to predict locoregional relapse and distant metastasis would be of great clinical value for improved survival and better prognosis.

Endemic NPC is strongly associated with Epstein‐Barr virus (EBV) infection.^4^ Cell‐free EBV DNA from NPC tumor cells can be detected in the plasma of NPC patients,^5^ and the plasma DNA load can reflect the tumor load and residual disease or subclinical metastases.^6^ Therefore, EBV DNA is widely considered as a reliable biomarker for early screening, clinical staging, prognosis prediction, individualized treatment, and follow‐up monitoring in NPC.^7^, ^8^, ^9^, ^10^, ^11^, ^12^, ^13^ While multiple studies have reported that plasma EBV DNA could be used as a predictive marker for NPC prognosis,^5^, ^14^, ^15^, ^16^, ^17^, ^18^ however, these studies only measured plasma EBV DNA load at limited time points, such as pretreatment and 3 months posttreatment. We suggested that a long‐term monitoring of dynamic changes of plasma EBV DNA could improve its prognostic value in NPC. In a meta‐analysis by Qu et al,^19^ plasma EBV DNA was measured primarily from day 1 to 3 months posttreatment. Therefore, the prognostic value of plasma EBV DNA at other time points, such as more than 3 months posttreatment, requires further study.

In addition, dynamic changes of plasma EBV DNA pre‐ and posttreatment may also be useful for prognosis prediction and treatment evaluation. Recently, several studies have reported the potential prognostic value of dynamic changes in plasma EBV DNA in NPC patients at different time points, including during treatment,^20^, ^21^ pre‐ and early posttreatment,^22^, ^23^ and within 3 months posttreatment.^24^, ^25^, ^26^ However, long‐term monitoring of dynamic changes in plasma EBV DNA after 3 months posttreatment might also provide prognostic value. Therefore, this study is to further examine the predictive value and dynamic changes of plasma EBV DNA load at multiple time points pre‐ and posttreatment in NPC.

## 2 MATERIALS AND METHODS

### 2.1 Patients

**FIGURE 1:**
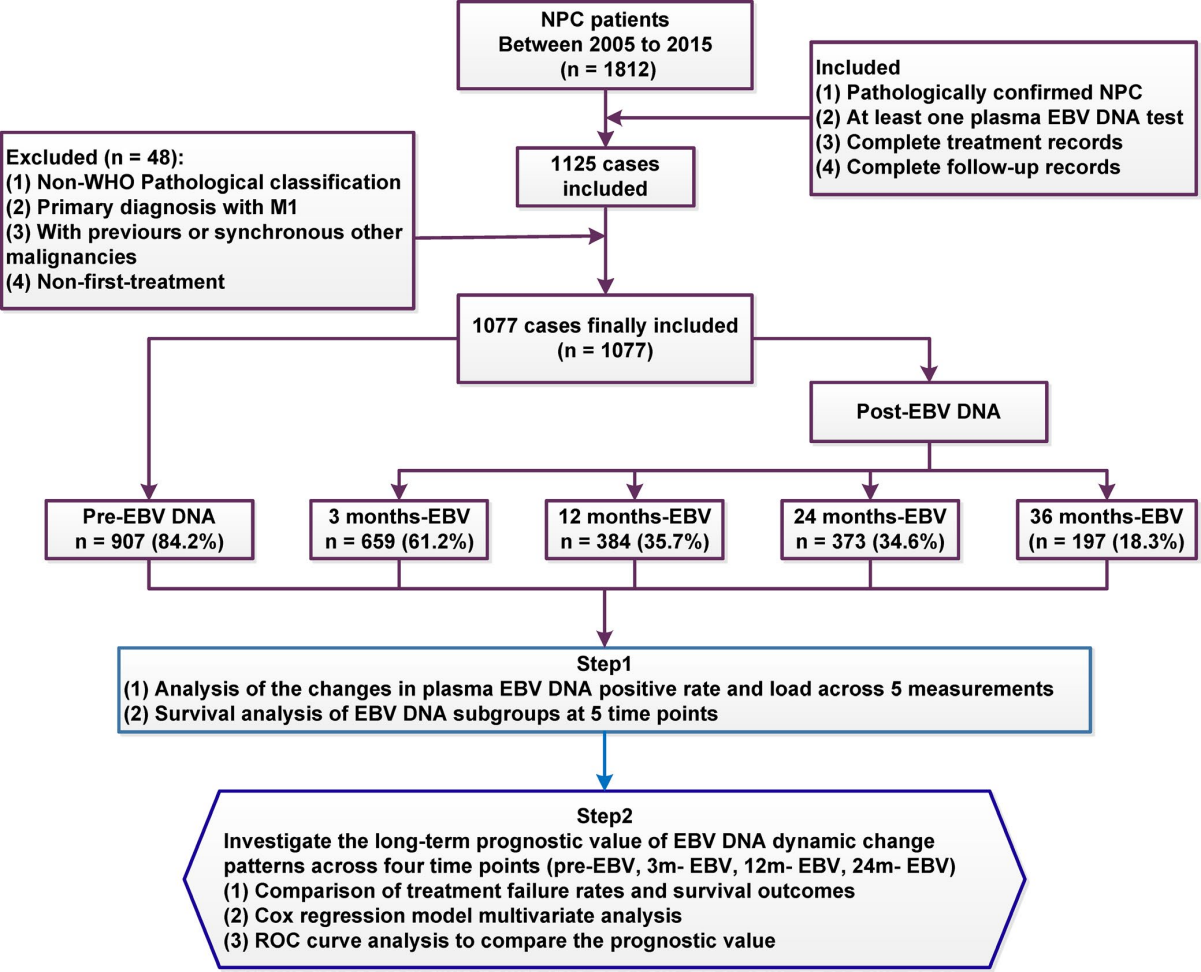
The flowchart of study design.

From January 2005 to December 2015, 1077 nonmetastatic NPC patients treated in Nanfang Hospital, Southern Medical University, were enrolled in this study. All cases were confirmed by pathological examinations and staged according to the 7th edition American Joint Committee on Cancer.^27^ The exclusion criteria consisted of non‐WHO pathological types, distant metastasis at initial diagnosis, prior malignancy, no plasma EBV DNA records. The work flow was shown in Figure 1. This study was approved by the Ethics Committee of Nanfang Hospital of Southern Medical University (Ethical review approval no.: NFEC‐2017‐165).

### 2.2 Treatment

All patients received intensity‐modulated radiotherapy (IMRT) at a total dose of 66–74 Gy for 6–8 weeks at target areas located by Computed Tomography (CT). In the patient cohort, 76 patients (7.1%) were at stage I and received IMRT treatment alone, 145 patients (13.4%) were at stage II and received concurrent chemoradiotherapy, and 856 patients (79.5%) were at stages III/IV and received concurrent chemoradiotherapy, induction chemotherapy, and/or adjuvant chemotherapy (Supporting Information S1).

### 2.3 Follow‐up

Patients were followed up at 3, 6, and 12 months in the first year after therapy, every 6 months during the second and third year, and yearly thereafter. Follow‐up evaluation included physical examination of the head and neck, nasopharyngeal endoscopy, chest radiography, abdominal ultrasound, EBV DNA serological testing, Magnetic Resonance Imaging (MRI) of nasopharynx and neck, and whole‐body PET. Patients with locoregional relapse and distant metastasis were confirmed by fine needle aspiration and pathological examination of biopsy. The primary study endpoint was progression‐free survival (PFS), and the secondary endpoints included distant metastasis‐free survival (DMFS), locoregional relapse‐free survival (LRFS), and overall survival (OS). (Supporting Information S2).

### 2.4 Quantification of plasma EBV DNA

Measurements of plasma EBV DNA were performed in the Laboratory Medicine Center of Nanfang Hospital, Southern Medical University. Venous blood samples (5 ml/each case) were collected before treatment and 3, 12 (±1 months), 24 (±3 months), and 36 (±3 months) months after treatment, placed in ethylenediaminetetraacetic acid (EDTA) tubes, and centrifuged at 1500× g for 5 min at 4°C. The plasma total DNA was extracted and EBV DNA concentration was determined by real‐time quantitative polymerase chain reaction (Supporting Information S3). After the PCR assay, samples with an undetectable EBV DNA signal were recorded as 0 copies/ml, and a positive plasma EBV DNA load was defined as >0 copies/ml. Referring to previous studies,^5^, ^16^, ^17^, ^28^, ^29^, ^30^, ^31^ the cutoff levels chosen to classify the patients into low and high EBV DNA groups were 1500 copies/ml pretreatment (low level group: <1500 copies/ml; high level group: ≥1500 copies/ml) and 0 copies/ml posttreatment (low level group: 0 copies/ml; high level group: >0 copies/ml) in this study.

### 2.5 Statistical analysis

Survival outcomes were estimated using the Kaplan‐Meier method and compared by the log‐rank test. The Cox proportional hazard model was used for multivariate analysis including the following variables: sex, age (≥45 years vs. <45 years), T stage (T4 vs. T1‐3), N stage (N2‐3 vs. N0‐1), pretreatment EBV DNA load (≥1500 copies/ml vs. <1500 copies/ml), and the patterns of dynamic change in plasma EBV DNA pre‐ and posttreatment. Receiver operating characteristic (ROC) curve analysis was performed to calculate the optimal cutoff value of plasma EBV DNA at each time point and compare the different prognostic values of dynamic change patterns and a single time point of plasma EBV DNA level. All statistical analyses were conducted using SPSS21.0 (IBM Corporation). A *p* < 0.05 (two tailed) was considered statistically significant for all tests.

## 3 RESULTS

### 3.1 Patient characteristics

The characteristics of 1077 NPC patients were listed in Table 1. The median follow‐up time was 54 months. During the long‐term follow‐up, 331 patients (30.7%) experienced disease progression, including 76 cases of locoregional recurrence (7.1%), 172 cases of distant metastasis (16.0%), 40 cases of both locoregional recurrence and distant metastasis (3.7%), and 147 deaths (13.6%, 104 patients died from locoregional recurrence or distant metastasis and 43 patients died without locoregional recurrence or distant metastasis). The 5‐year PFS, DMFS, LRFS, and OS rates were 68.4%, 79.2%, 87.7%, and 84.8%, respectively.

**TABLE 1:** Clinical characteristics of NPC patients (*n* = 1077)

Characteristic	*N* (%)
Sex	
Female	284 (26.4)
Male	793 (73.6)
Age (years)	
<45	469 (43.5)
≥45	608 (56.5)
Smoking	
Yes	657 (61.0)
No	420 (39.0)
WHO pathologic type^a^	
Keratinizing carcinoma	6 (0.6)
Differentiated non‐keratinizing carcinoma	79 (7.3)
Undifferentiated non‐keratinizing carcinoma	992 (92.1)
Overall stage^b^	
I	76 (7.1)
II	145 (13.5)
III	344 (31.9)
IV	512 (47.5)
Tumor stage^b^	
T1	222 (20.6)
T2	213 (19.8)
T3	205 (19.0)
T4	437 (40.6)
Node stage^b^	
N0	162 (15.1)
N1	311 (28.9)
N2	512 (47.5)
N3	92 (8.5)

Abbreviation: NPC, nasopharyngeal carcinoma.

^a^ Pathologic type according to the 2005 World Health Organization (WHO) classification of tumors.

^b^ According to the 7th edition of the UICC/AJCC staging system.

### 3.2 Pre‐ and posttreatment plasma EBV DNA levels at various time points

**FIGURE 2:**
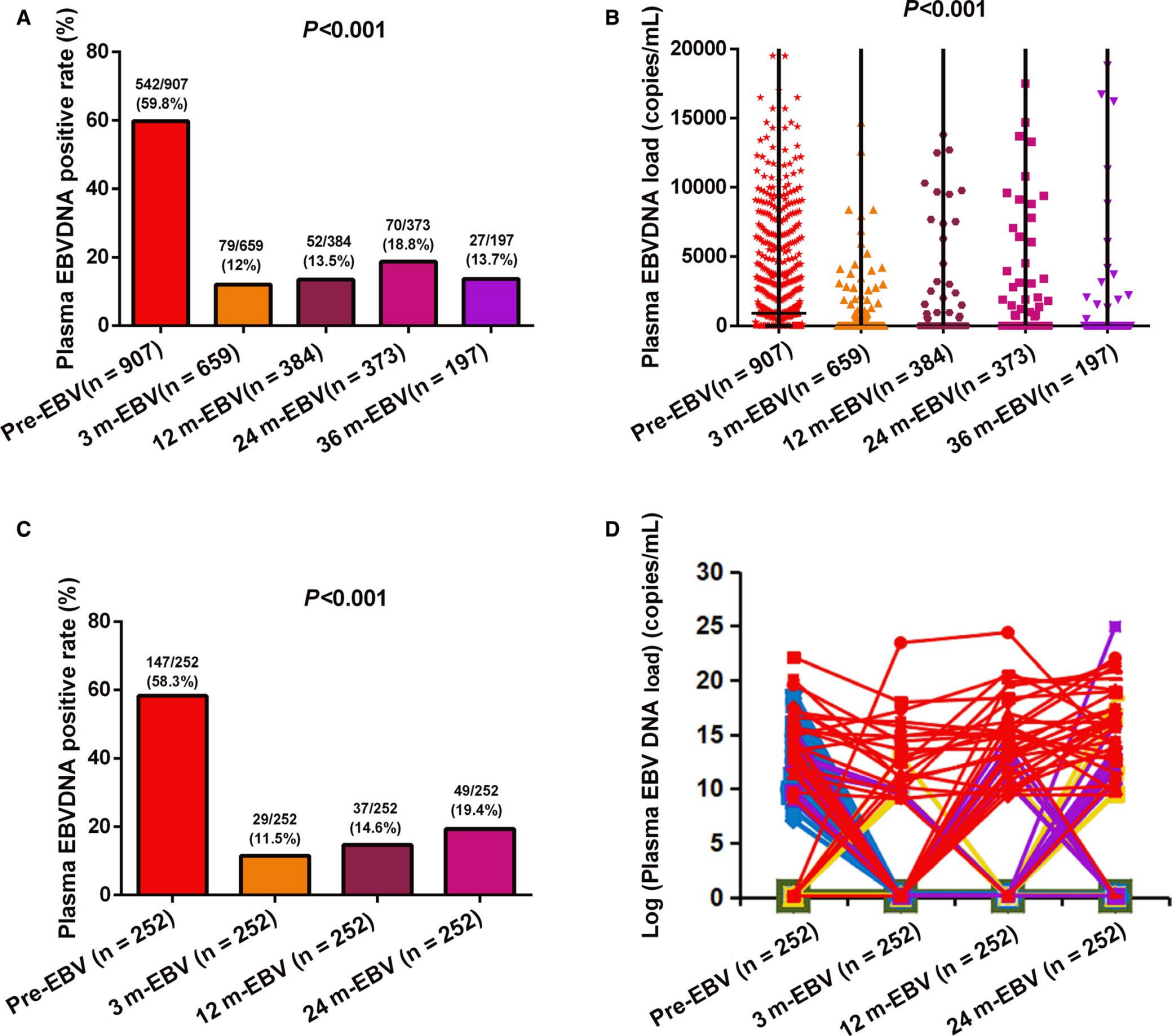
The positive rate and viral load of plasma Epstein‐Barr virus (EBV) DNA pre‐ and posttreatment. (A) Comparisons of the positive rates of plasma EBV DNA at all five time points among 1077 nasopharyngeal carcinoma (NPC) patients. (B) Comparisons of plasma EBV DNA copy numbers at all five time points among 1077 NPC patients. (C) Comparisons of the positive rates of plasma EBV DNA at 4 time points pre‐ and posttreatment among 252 NPC patients receiving four tests. (D) Comparisons of viral load of plasma EBV DNA at four time points pre‐ and posttreatment among 252 NPC patients receiving four tests.

A total of 1077 patients received plasma EBV DNA tests at a single time point pre‐ and posttreatment (Figure 1), and 252 patients received plasma EBV DNA tests at four time points (pretreatment, 3, 12, and 24 months posttreatment). The results showed that the positive rate and viral load of pretreatment plasma EBV DNA were significantly higher than those at each time point posttreatment (Figure 2A,B). Among 252 patients receiving plasma EBV DNA tests at four time points pre‐ and posttreatment, posttreatment EBV‐DNA positivity was significantly lower than pretreatment, and posttreatment plasma EBV DNA seemed to show an increase over time (Figure 2C). As shown in Figure 2D, the changes of plasma EBV DNA load pre‐ and posttreatment were quite different among these 252 NPC patients. Some patients maintained plasma EBV DNA baseline negativity (0 copies/ml) at all four time points, some patients showed plasma EBV DNA above baseline at only a single time point (pre‐ or posttreatment), and some patients rebounded above the baseline level after treatment (single or multiple time points), while some patients maintained plasma EBV DNA positivity at all four time points.

**FIGURE 3:**
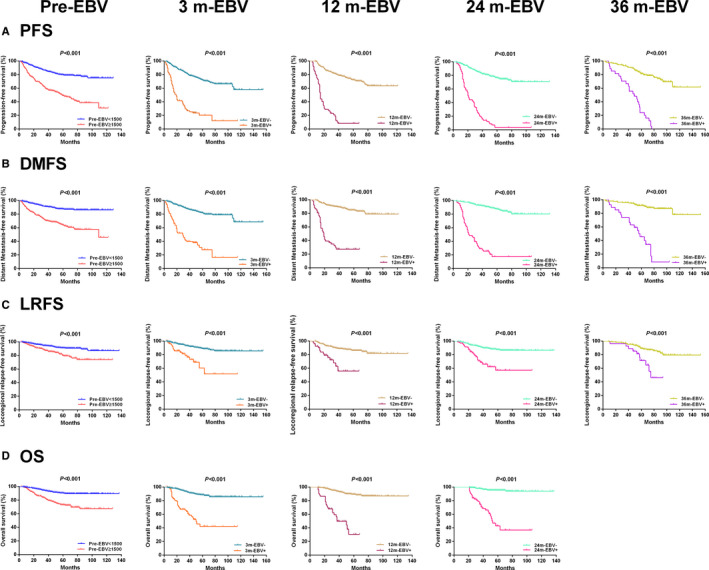
Kaplan‐Meier plots of survival outcomes for subgroups at different time points. (A) Progression‐free survival. (B) Distant metastasis‐free survival. (C) Locoregional relapse‐free survival. (D) Overall survival.

Patients with pretreatment plasma EBV DNA load ≥1500 copies/ml had worse 5‐year PFS, DMFS, LRFS, and OS than those with plasma EBV DNA load <1500 copies/ml (all *p* < 0.001). Patients with plasma EBV DNA levels >0 copies/ml at three different time points posttreatment (3, 12, and 24 months) had a significantly lower 5‐year PFS, DMFS, LRFS, and OS than those with negative plasma EBV DNA (all *p* < 0.001). There were also significant differences in 5‐year PFS, DMFS, and LRFS between patients with EBV DNA levels >0 copies/mL and EBV DNA levels <0 copies/ml at 36 months posttreatment (all *p* < 0.001; Table S1; Figure 3). However, it was difficult to compare OS between these two groups at 36 months posttreatment as there was only one death among these patients.

### 3.3 Dynamic changes in plasma EBV DNA at 4 time points pre‐ and posttreatment

The positive rate and viral load of plasma EBV DNA fluctuated markedly at the 4 time points pre‐ and posttreatments (Figure 2C,D). To examine the prognostic value of dynamic changes in plasma EBV DNA, we divided the 252 patients receiving tests at all four time points (pretreatment and 3, 12, and 24 months posttreatment) into five subgroups according to the dynamic change pattern observed in Figure 2D. Pattern 1 (Pre− & Post 0+) consisted of 82 patients (82/252, 32.5%) with persistently undetectable plasma EBV DNA at all four time points. Pattern 2 (Pre+ & Post 0+) included 104 patients (104/252, 41.3%) with positive detection pretreatment but negative detection at all‐time points posttreatment. Pattern 3 (Pre− & Post 1+) included 18 patients (18/252, 7.2%) with negative detection pretreatment and positive detection at one time point posttreatment. Pattern 4 (Pre+ & Post 1+) contained 17 patients (17/252, 6.7%) with positive detection pretreatment and at one time point posttreatment. Pattern 5 (Pre−/+ & Post ≥2+) included 31 patients (31/252, 12.3%) with positive detection at ≥2 time points posttreatment and negative or positive detection pretreatment (Table 2).

**TABLE 2:** Comparisons of 3‐year survival rates among groups defined by plasma EBV DNA dynamic change patterns across 4 time points pre‐ and posttreatment

Subject	*N* (%)	PFS	DMFS	LRFS	OS
EBV‐DNA Dynamic change pattern	252	*p* < 0.001	*p* < 0.001	*p* < 0.001	*p* < 0.001
Pattern 1 (Pre‐ & Post 0+)	82 (32.5%)	91.4%	97.5%	95.0%	96.2%
Pattern 2 (Pre+ & Post 0+)	104 (41.3%)	86.2%	92.0%	92.1%	99.0%
Pattern 3 (Pre‐ & Post 1+)	18 (7.2%)	66.7%	77.8%	88.2%	94.4%
Pattern 4 (Pre+ & Post 1+)	17 (6.7%)	39.7%	70.6%	69.5%	87.4%
Pattern 5 (Pre‐/+ & Post ≥2+)	31 (12.3%)	6.5%	17.9%	49.1%	55.3%

Abbreviations: DMFS, distant metastasis‐free survival; LRFS, locoregional relapse‐free survival; OS, overall survival; PFS, progression‐free survival.

**FIGURE 4:**
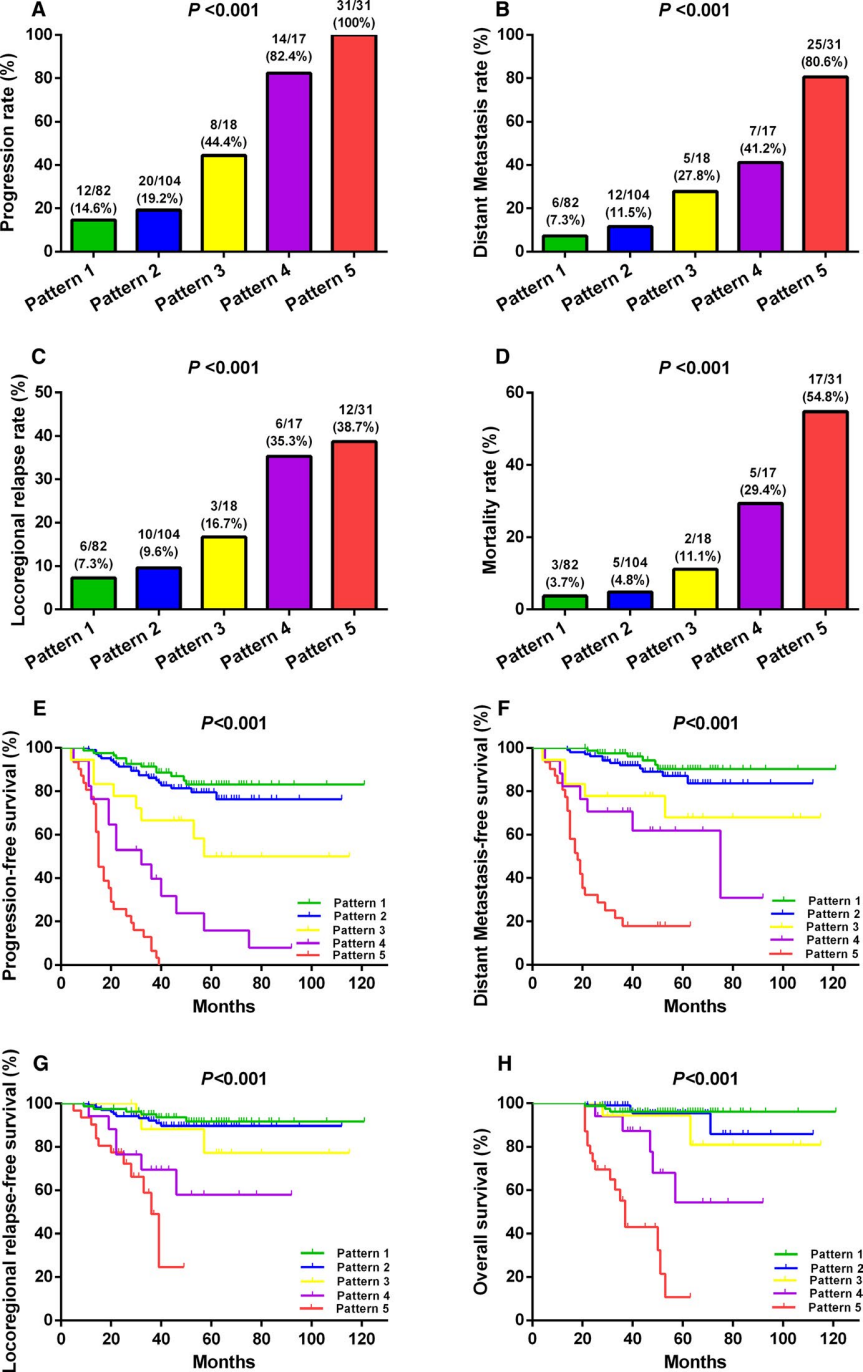
Comparisons of treatment failure rates and survive outcomes among five different patterns defined by dynamic changes in plasma Epstein‐Barr virus DNA pre‐ and posttreatment. (A) Progression rate. (B) Distant metastasis rate. (C) Locoregional relapse rate. (D) Mortality rate. (E) Progression‐free survival. (F) Distant metastasis‐free survival. (G) Locoregional relapse‐free survival. (H) Overall survival.

There were significant differences in the rates of disease progression, distant metastasis, locoregional relapse, and mortality among these five patterns (all *p* < 0.001; Figure 4A‐D). Similarly, the 3‐year PFS, DMFS, LRFS, and OS also differed significantly among these five patterns (all *p* < 0.001; Figure 4E‐H). The patients of Pattern 1 had the best prognosis, followed by Patterns 2, 3, and 4, while the patients of Pattern 5 had the worst prognosis (Table 2; Figure 4).

### 3.4 Multivariate analysis using a Cox hazard ratio model

Multivariate analysis identified the pattern of dynamic changes in plasma EBV DNA (defined by four time points pre‐ and posttreatment) as an independent predictor of PFS, DMFS, LRFS, and OS in NPC patients (Table 3). Pattern 4 and Pattern 5 were independent risk factors for worse PFS, DMFS, LRFS, and OS (all *p* < 0.05), however, Pattern 3 was an independent risk factor for poorer PFS and DMFS (all *p* < 0.01).

**TABLE 3:** Multivariate analysis of prognostic risk factors in NPC patients receiving four plasma EBV DNA tests (*n* = 252)

Variable	PFS		DMFS		LRFS		OS	
	HR (95% CI)	*p* value	HR (95% CI)	*p* value	HR (95% CI)	*p* value	HR (95% CI)	*p* value
Sex (male vs. female)	1.828 (1.001–3.337)	0.050	2.079 (0.977–4.425)	0.057	0.578 (0.241–1.385)	0.219	2.213 (0.689–7.112)	0.182
Age (≥45 vs. <45)	0.988 (0.635–1.536)	0.957	0.783 (0.448–1.367)	0.389	0.929 (0.477–1.808)	0.828	1.220 (0.588–2.530)	0.594
Smoking (Yes vs. No)	0.764 (0.458–1.274)	0.302	0.588 (0.310–1.116)	0.104	1.592 (0.714–3.550)	0.256	0.977 (0.431–2.217)	0.956
T stage (T4 vs. T1‐3)	2.071 (1.308–3.277)	0.002	2.268 (1.261–4.077)	0.006	1.301 (0.665–2.542)	0.442	1.788 (0.837–3.822)	0.134
N stage (N2‐3 vs. N0‐1)	1.192 (0.706–2.012)	0.511	1.767 (0.845–3.698)	0.131	0.872 (0.412–1.844)	0.719	1.269 (0.485–3.321)	0.628
Pre‐EBV (≥1500 vs. <1500)	0.737 (0.401–1.354)	0.325	0.667 (0.315–1.411)	0.290	1.624 (0.607–4.343)	0.334	0.636 (0.256–1.583)	0.331
EBV DNA Dynamic change pattern		<0.001		<0.001		<0.001		<0.001
Pattern 1 (Pre‐ & Post 0+)	Reference	Reference	Reference	Reference	Reference	Reference	Reference	Reference
Pattern 2 (Pre+ & Post 0+)	1.436 (0.636–3.244)	0.384	1.847 (0.625–5.454)	0.267	0.919 (0.256–3.297)	0.897	1.617 (0.345–7.588)	0.542
Pattern 3 (Pre‐ & Post 1+)	4.296 (1.699–10.863)	0.002	6.591 (1.910–22.744)	0.003	2.176 (0.534–8.878)	0.278	3.403 (0.546–21.225)	0.190
Pattern 4 (Pre+ & Post 1+)	10.409 (4.043–26.794)	<0.001	7.865 (2.242–27.593)	0.001	5.131 (1.213–21.708)	0.026	11.076 (2.238–54.823)	0.003
Pattern 5 (Pre‐/+ & Post ≥2+)	30.637 (12.456–75.355)	<0.001	31.581 (10.154–98.220)	<0.001	8.176 (2.193–30.485)	0.002	43.442 (9.850–191.603)	<0.001

Abbreviations: DMFS, distant metastasis‐free survival; LRFS, locoregional relapse‐free survival; NPC, nasopharyngeal carcinoma; OS, overall survival; PFS, progression‐free survival.

### 3.5 ROC curve analysis

**FIGURE 5:**
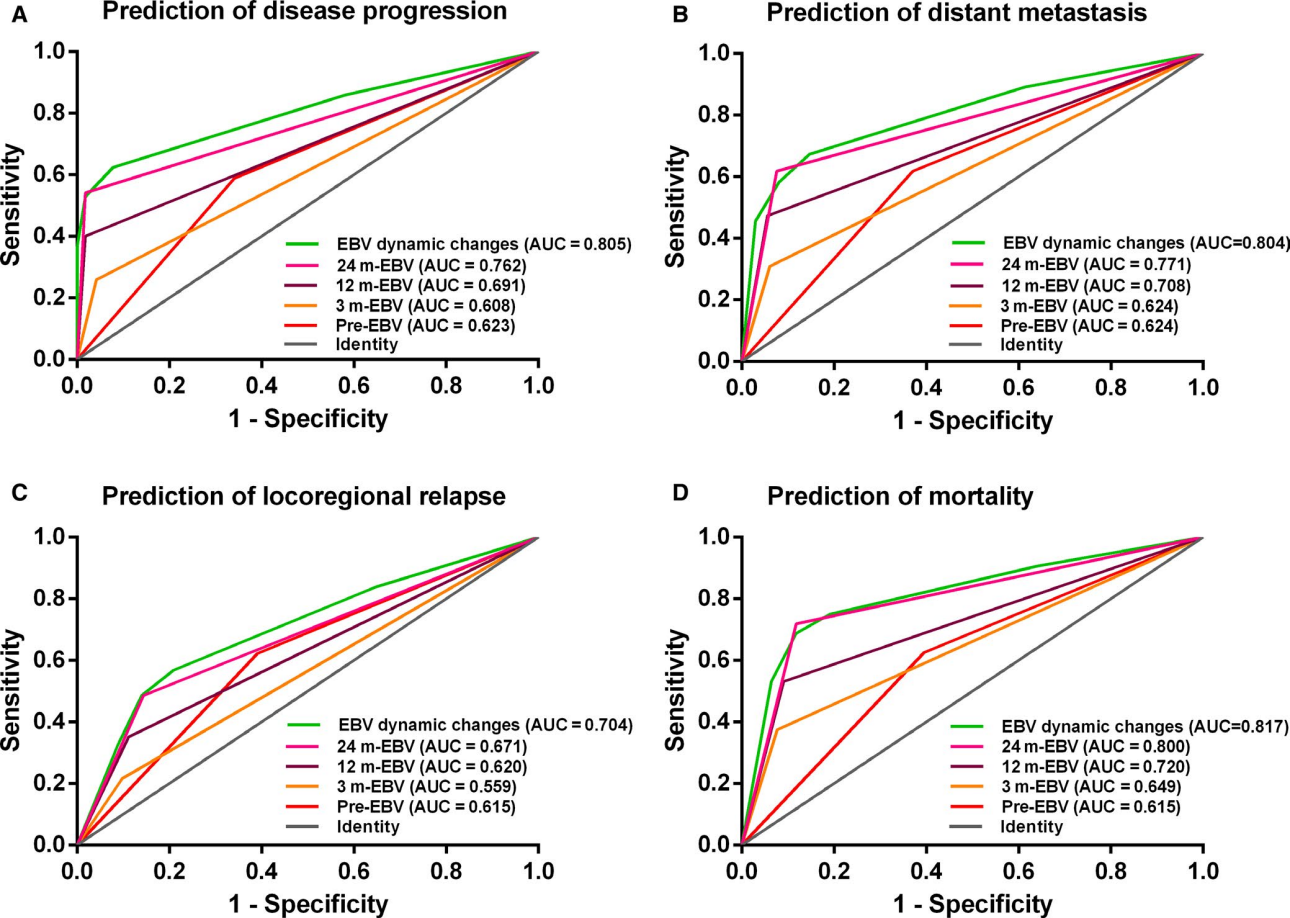
Receiver operating characteristic curve analysis in comparing the prognostic value of dynamic change patterns and a single time point of plasma Epstein‐Barr virus (EBV) DNA level. (A)Prediction of disease progression. (B) Prediction of distant metastasis. (C) Prediction of locoregional relapse. (D) Prediction of mortality.

Receiver operating characteristic analyses demonstrated that dynamic changes in plasma EBV DNA showed larger area under the curve (AUC) values than plasma EBV DNA level at any single time point for predicting NPC disease progression (AUC = 0.805; 95% CI, 0.740–0.869), distant metastasis (AUC = 0.804; 95% CI, 0.730–0.879), locoregional relapse (AUC = 0.704; 95% CI, 0.606–0.802), and mortality (AUC = 0.817; 95% CI, 0.726–0.908) (all *p* < 0.001) (Figure 5).

## 4 DISCUSSION

Multiple reports have shown that pre‐ and posttreatment plasma EBV DNA are prognostic factors for NPC progression and survival,^5^, ^14^, ^15^, ^16^, ^17^, ^18^ and these findings are also confirmed in this study. However, these previous studies mainly focused on few time points, for example, pretreatment and 3 months posttreatment.^19^ In this study, we investigated the prognostic value of plasma EBV DNA load at multiple time points during a long‐time follow‐up, and examined the patterns of dynamic changes in plasma EBV DNA across 4 time points (pretreatment, 3, 12, and 24 month posttreatment). We found that Dynamic changes in plasma EBV DNA pre‐ and posttreatment could predict the long‐term survival outcome and provide accurate risk stratification in NPC.

In this study, we found that positive rate of plasma EBV DNA decreased significantly posttreatment, but increased again slowly over time, with a peak at 24 month posttreatment (Figure 2C). Comparisons of 5‐year survival rates at different time points pre‐ and posttreatment showed that the differences of PFS and DMFS were bigger at 24 month posttreatment than at several other time points. This was consistent with Liu et al^32^ and Wu et al,^33^ who found a sharp peak in the risk curve of treatment failure and NPC‐related death at 2 years posttreatment. Plasma EBV DNA level at 2 years posttreatment appeared to be critical for disease surveillance of NPC patients. Furthermore, this peak in treatment failure risk coincided with the peak of disease progression observed posttreatment seen in our daily clinical practice. Therefore, monitoring and evaluation of plasma EBV DNA was particularly important at 2 years posttreatment, which could effectively predict disease progression and treatment failure.

Many studies have reported that plasma EBV DNA loads and its dynamic changes could be used as a predictive marker for NPC prognosis at limited time points,^20^, ^21^, ^22^, ^23^, ^24^, ^25^, ^26^ such as pretreatment and 3 months posttreatment, however, the prognostic value and dynamic changes of plasma EBV DNA at other time points, such as more than 3 months posttreatment, are not be fully understood yet. Notably, in this study, we enrolled 252 NPC patients who received plasma EBV DNA tests at 4 time points (pretreatment, 3, 12, and 24 months posttreatment). We showed the dynamic changes in plasma EBV DNA at four different time points. Based on this dynamic change, we divided 252 patients into five subgroups/patterns and performed survival analysis. This was different from the previous reports,^20^, ^21^, ^22^, ^23^, ^24^, ^25^, ^26^ which mainly focused on the following time points primarily from pretreatment to 3 months posttreatment.

We found that there was significant difference on prognosis among five patterns of dynamic changes in plasma EBV DNA, which suggested that the more positive time points of plasma EBV DNA detection, the higher the risk of disease progression, distant metastasis, locoregional recurrence and death. The patients of pattern 5 had the worst prognosis on progression, metastasis, recurrence and mortality, and the patients of pattern 1 had the best prognosis. This conclusion was similar to other studies.^34^, ^35^ As to pattern 2 and pattern 3, the patients of pattern 2 had a better prognosis than those of pattern 3, consistent with Qu's meta‐analysis that posttreatment EBV DNA level had greater impact than pretreatment EBV DNA on clinical outcomes of NPC.^19^ Moreover, the patients of pattern 4 had a worse prognosis than those of pattern 2 and 3, the possible explanation was that these patients had a higher baseline and residual tumor load.^15^

The results of multivariate analysis showed that dynamic change in plasma EBV DNA pre‐ and posttreatment was an independent prognostic biomarker for NPC patients (*p* < 0.001). Compared with pattern 1, the risk stratification of disease progression, distant metastasis, locoregional relapse, and mortality were gradually increased from pattern 2 to pattern 5, while the hazard ratio of disease progression, distant metastasis, locoregional relapse and mortality of pattern 5 were as high as 30.637, 31.581, 8.176, and 43.442, respectively. Therefore, patterns of dynamic changes in plasma EBV DNA pre‐ and posttreatment could provide more accurate and in‐depth information for risk stratification of NPC patients and helps to distinguish patients with poor prognosis for further treatment.

In daily clinical practice, it is very important to early detect locoregional tumor recurrence posttreatment, because timely salvage treatment could improve survival outcome of these patients.^36^ Chen's findings showed that positive cell free EBV DNA results preceded radiological and/or clinical evidence of disease recurrence by a median of 2.3 months.^35^ These findings confirmed that plasma EBV DNA was a significant sensitive and noninvasive predictor of tumor recurrence. Based on our results, we suggested a long‐term follow‐up program according to dynamic changes in plasma EBV DNA. We could examine plasma EBV DNA load and evaluate the risk of disease progression, distant metastasis, locoregional recurrence and mortality. The patients with positivity detection of plasma EBV DNA at any time point posttreatment need to be adjusted to a closer follow‐up surveillance strategy, while patients with positive plasma EBV DNA at two or more points posttreatment should be give much attention, and necessary radiological and/or physical examinations should be performed to exclude locoregional recurrence or distant metastasis.

This study also has several limitations. First, the retrospective design might introduce selection bias. Second, the sample size (252 cases) has been the largest to date for assessment of dynamic changes in plasma EBV DNA over multiple time points, however, it was still insufficient for investigating more complex change patterns. Finally, the long‐term follow‐up program we proposed cannot be further verified in this study. Therefore, a prospective, multicenter, randomized clinical trial with larger sample sizes for a long‐term follow‐up are needed in future.

## 5 CONCLUSIONS

Plasma EBV DNA load at multiple time points pre‐ and posttreatment (up to 36 months) was a significant predictor for NPC prognosis, and the dynamic change pattern in plasma EBV DNA at these time points could provide much accurate predictive value for NPC prognosis and risk stratification. Therefore, serial plasma EBV DNA measurements extending for several years posttreatment and assessment of dynamic changes in plasma EBV DNA load should be conducted routinely in clinical practice to identify high‐risk patients for further treatment.

## CONFLICT OF INTEREST

The authors declare that they have no competing interests.

## AUTHOR CONTRIBUTIONS

Study concept: Xiong Liu. Study design: Juan Lu and Feipeng Zhao. Data acquisition: Wanxia Li, Jing Chen, and Bijun Liang. Quality control of data and algorithms: Fangfang Zeng and Xinyu Peng. Data analysis and interpretation: Zonghua Li, Junzheng Li, Xiaofei Yuan, and Shuting Wu. Statistical analysis: Yanfei Li, Junzheng Li, and Xiaofei Yuan. Article preparation: Wanxia Li, Jing Chen, and Bijun Liang. Article editing: Juan Lu and Feipeng Zhao. Article review: All authors.

## Supporting information

Supplementary Material

## Data Availability

All data included in this study are available upon request by contact with the corresponding author.
