# Supplementary material for: Long‐term monitoring of dynamic changes in plasma EBV DNA for improved prognosis prediction of nasopharyngeal carcinoma
Source: Cancer Med. 2020 Dec 30;10(3):883–94. doi: 10.1002/cam4.3669 (PMC7897970; doi:10.1002/cam4.3669)
Supplement: Supplementary file 1 — Supplementary Material [file CAM4-10-883-s001.docx]

**Supplementary Materials**

**Supplementary Material 1** Detailed treatment protocols for NPC patients

(i) Radiotherapy

All patients in this study received intensity-modulated radiotherapy(IMRT) to targets located by CT. The irradiation doses were as follows. The total dose to the planning target volume (PTV) in the primary tumor area (GTVnx) was 70-74 Gy, that to the neck metastatic lymph node area (GTVnd) was 66-70 Gy, that to the high-risk clinical target volume (CTV1) was 60-62 Gy, and that to the low-risk clinical target volume (CTV2) was 50-56 Gy. The total dose was divided into 28 and 33 applications delivered once per day, 5 days per week, at a single dose of 2.12-2.24 Gy.

(ii) Chemotherapy

All chemotherapy regimens were cisplatin-based combination regimens and a complete cycle was administered every 3 weeks. The induction chemotherapy regimen (1 or 2 cycles) and adjuvant chemotherapy regimen (1-4 cycles) included TP (docetaxel 60 mg/m^2^/d or paclitaxel 135 mg/m^2^/d on day 1 and cisplatin 25 mg/m^2^/d on days 1 to 3), TPF (docetaxel 60 mg/m^2^/d or paclitaxel 135 mg/m^2^/d on day 1, cisplatin 25 mg/m^2^/d on days 1 to 3, and 5-fluorouracil 600 mg/m^2^/d on days 1 to 5), and PF (cisplatin 25 mg/m^2^/d on days 1 to 3 and 5-fluorouracil 600 mg/m^2^/d on days 1 to 5). The concurrent chemotherapy regimens (1 or 2 cycles) included cisplatin monotherapy (once every three weeks, 25 mg/m^2^/d on days 1 to 3) and TP (docetaxel 60 mg/m^2^/d or paclitaxel 135 mg/m^2^/d on day 1 and cisplatin 25 mg/m^2^/d on days 1 to 3).

**Supplementary Material 2** The definition of the study endpoints

The primary study endpoint was progression-free survival (PFS), which was defined as the time from the initial pathological diagnosis of NPC to relapse at any site or death from any cause, whichever occurred first, or last follow-up visit. The secondary endpoints included distant metastasis-free survival (DMFS), locoregional relapse-free survival (LRFS), and overall survival (OS). DMFS was defined as the time from pathological diagnosis to distant metastasis detection, death, or last follow-up visit. LRFS was defined as the time from pathological diagnosis to relapse in nasopharynx or neck lymph nodes, death, or last follow-up visit. OS was defined as the time between initial pathological diagnosis of NPC and all-cause death or last follow-up visit.

**Supplementary Material 3** Plasma EBV DNA measurements

Plasma EBV DNA measurements were completed in the Laboratory Medicine Center of Nanfang Hospital, Southern Medical University. Venous blood samples (5 ml/each case) were collected before treatment and 3, 12, 24, and 36 months after treatment, placed in ethylenediaminetetraacetic acid (EDTA) tubes, and centrifuged at 1500×g for 5 min at 4°C. Plasma total DNA was extracted using the QIAamp blood kit (Qiagen, Hilden, Germany). The BamH I-W region of the EBV genome was amplified by real-time quantitative PCR (RT-qPCR) using an EBV RT-qPCR kit and primers 5′-GCTGCGCTGCTGCTATCTT-3′ (forward) and 5′-CAAGCCCACTCCCCTGTCT-3′ (reverse) according to the manufacturer’s instructions (Liferiver, Shanghai, China). The GAPDH gene was amplified as an internal control using the primers 5′-GGCGACGCAAAAGAAGATG-3′ (forward) and 5′-CCGTTGACTCCGACCTTCAC-3′ (reverse). PCR conditions were as follows: initial denaturation at 95°C for 10 minutes, followed by 40 cycles of denaturation at 95°C for 15 seconds and amplification at 56°C for 1 minute. The EBV DNA copy number was calculated according to the formula C = Q × (V_DNA_/V_PCR_) × (1/V_EXT_), where C represented the target concentration in plasma (copies/ml), Q meant the target quantity (copies) detected by PCR, V_DNA_ meant the DNA total volume obtained after extraction, V_PCR_ meant the volume of the DNA solution used for PCR amplification, and V_EXT_ meant the volume of plasma extracted.

**Supplementary Table 1** Comparisons of 5-year survival rates among NPC patient groups defined by plasma EBV DNA status at 5 time points before and after treatment

| Time points and groups | N (%) | Survival rate | | | |
| --- | --- | --- | --- | --- | --- |
|  |  | PFS | DMFS | LRFS | OS |
| Pre-treatment EBV | 907 (84.2%) | *P*<0.001 | *P*<0.001 | *P*<0.001 | *P*<0.001 |
| <1500 | 541 (59.6%) | 79.9% | 87.7% | 91.1% | 90.5% |
| ≥1500 | 366 (40.4%) | 49.3% | 64.1% | 80.1% | 73.3% |
| 3 months post-treatment EBV | 659 (61.2%) | *P*<0.001 | *P*<0.001 | *P*<0.001 | *P*<0.001 |
| undetectable (0 copies/mL) | 580 (88.0%) | 72.3% | 82.0% | 89.1% | 88.6% |
| Detectable (>0 copies/mL) | 79 (12.0%) | 20.3% | 27.6% | 60.2% | 42% |
| 12 months post-treatment EBV | 384 (35.7%) | *P*<0.001 | *P*<0.001 | *P*<0.001 | *P*<0.001 |
| undetectable (0 copies/mL) | 332 (86.5%) | 72.3% | 84.9% | 86.1% | 89.4% |
| Detectable (>0 copies/mL) | 52 (13.5%) | 8.4% | 26.9% | 55.4% | 29.7% |
| 24 months post-treatment EBV | 373 (34.6%) | *P*<0.001 | *P*<0.001 | *P*<0.001 | *P*<0.001 |
| undetectable (0 copies/mL) | 303 (81.2%) | 76.4% | 87.5% | 87.3% | 95.5% |
| Detectable (>0 copies/mL) | 70 (18.8%) | 3.5% | 17.5% | 56.8% | 42.7% |
| 36 months post-treatment EBV | 197 (18.3%) | *P*<0.001 | *P*<0.001 | *P*<0.001 | --- |
| undetectable (0 copies/mL) | 170 (86.3%) | 81.3% | 91.5% | 89.0% | --- |
| Detectable (>0 copies/mL) | 27 (13.7%) | 24.1% | 45.6% | 71.3% | --- |
| Abbreviations: PFS=progression-free survival; DMFS=distant metastasis-free survival; LRFS=locoregional relapse-free survival; OS=Overall survival. | | | | | |
